# Participation of adolescents from the *Quilombola* community in the creation of an educational game about alcohol consumption

**DOI:** 10.1590/1980-220X-REEUSP-2021-0402

**Published:** 2022-04-01

**Authors:** Adriana Nunes Moraes-Partelli, Marta Pereira Coelho, Séfora Gasparini Santos, Isabela Lorencini Santos, Ivone Evangelista Cabral

**Affiliations:** 1 Universidade Federal do Espírito Santo, Centro Universitário Norte do Espírito Santo, Departamento de Ciências da Saúde, São Mateus, ES, Brazil.; 2 Universidade Federal do Rio de Janeiro, Escola de Enfermagem Anna Nery, Programa Pós-Graduação em Enfermagem, Rio de Janeiro, Brazil.

**Keywords:** Educational and Promotional Materials, Adolescent, Ethanol, African Continental Ancestry Group, Pediatric Nursing, Materiais Educativos e de Divulgação, Adolescente, Álcool, Grupo com Ancestrais do Continente Africano, Enfermagem Pediátrica, Materiales Educativos y de Divulgación, Adolescente, Etanol, Grupo de Ascendencia Continental Africana, Enfermería Pediátrica

## Abstract

**Objective:**

To describe and discuss the participation of adolescents from a *quilombola* community in the transformation of the comic “Possible Story” (“*Uma História Possível*”), from the Comic on alcohol, into an educational game.

**Method:**

Implementation of the creative and sensitive method of art-based research, with adolescents from a *quilombola* community in the state of Espírito Santo, for the development of a board game.

**Results:**

The democratic and interactive space favored the problematization of images and narratives about alcohol consumption mediated in the comic book. The group selected scenes, reordered the story, devised questions and riddles, formulated true and false assertions in a 17-card composition of the board game. The potential of the game as a content mediating tool to promote learning, reinforcement, and fixation of scientific content was evaluated.

**Conclusion:**

The active and dynamic participation of adolescents took place from conception to evaluation of the board game, encouraging them to reflect on a community context of cultural permissiveness of alcohol use.

## RESUMO

**Objetivo:**

Descrever e discutir a participação de adolescentes de uma comunidade quilombola na transformação de quadrinhos de “Uma História Possível”, do Almanaque sobre o álcool, em jogo educativo.

**Método:**

Implementação do método criativo e sensível de pesquisa baseada em arte, com adolescentes de uma comunidade quilombola do Espírito Santo, no desenvolvimento de um jogo de tabuleiro.

**Resultados:**

O espaço democrático e interativo favoreceu a problematização de imagens e narrativas sobre o consumo de álcool mediadas na história em quadrinhos. O grupo selecionou cenas, reordenou a história, elaborou perguntas e charadas, formulou asserções de verdadeiro e falso numa S composição de 17 cartas do jogo de tabuleiro. Avaliou-se o potencial do jogo como ferramenta mediadora de conteúdo para promover a aprendizagem, o reforço e fixação de conteúdos científicos.

**Conclusão:**

A participação ativa e dinâmica de adolescentes deu-se desde a concepção até a avaliação do jogo de tabuleiro, estimulando-os à reflexão sobre um contexto comunitário de permissividade cultural de uso do álcool.

## RESUMEN

**Objetivo:**

Describir y discutir la participación de adolescentes de una comunidad *quilombola* en la transformación de cómics de “Una Historia Posible” (*Uma História Possível*), del Almanaque sobre alcohol en juego educativo.

**Método:**

Implementación del método creativo y sensible de investigación basada en arte, con adolescentes de una comunidad *quilombola* de Espírito Santo, Brasil, en el desarrollo de un juego de mesa.

**Resultados:**

El espacio democrático e interactivo favoreció la problematización de imágenes y narrativas sobre el consumo de alcohol mediado en los cómics. El grupo seleccionó escenas, reordenó la historia, elaboró preguntas y charadas, formuló aserciones de mito o verdad en una composición de 17 fichas del juego de mesa. Se evaluó el potencial del juego como herramienta mediadora de contenido para promover el aprendizaje, el repaso y la memorización de contenidos científicos.

**Conclusión:**

La participación activa y dinámica de adolescentes ocurrió desde la concepción hasta la evaluación del juego de mesa, estimulándolos a la reflexión sobre un contexto comunitario de permisividad cultural de uso del alcohol.

## INTRODUCTION

Early use and consumption of alcohol by adolescents is associated with behavioral and social problems and risk of substance dependence^(1)^. According to data from the 2015 National School Health Survey (*PeNSE*), 55.5% of elementary school students aged 13 to 15 years have tried some alcoholic beverage. The experience of alcoholic intoxication at least once corresponded to 21.4%; 7.3% of adolescents had problems with family or friends, skipped classes, or got involved in fights after drinking alcohol^(2)^.

For many adolescents, the first experiences with alcohol take place in the family environment, in the presence of parents, and with their support^(3)^. In a *quilombola* community in the north of Espírito Santo, alcohol consumption is culturally and socially more accepted among men than women. In this consumption, the intergenerational lineage involves grandparents, the father, and the oldest children^(4)^.

In that *quilombola* community, alcohol is consumed in all private and public spaces, including family celebrations, sports games, or community parties. The exposure image of a permissive consumption leads adolescent boys and girls to the naturalization of drinking as part of the rites of passage and the construction of their masculinities and femininities. Living in this environment becomes a reference in the process of socialization of adolescents while representing a challenge for health workers in the approach to health promotion and education with this social group^(4)^. 

In this context, the game emerges as a valuable educational health technology for the development of health education. The game is a playful resource that excites students, favors the memorization of information, and stimulates interaction in the different moments of the teaching-learning process^(5)^. 

From this perspective, it is understood that the game, as educational material, may be suitable to address health issues, with emphasis on the characteristics of the participatory development process of the public to whom this material is intended^(6)^, as is the case of adolescents of Afro-descendant matrices. In the experience of adolescents from *quilombola* communities, participating in the construction of an educational game gathering local and scientific knowledge about alcohol can become a facilitator for discussion and reflection on a sensitive topic that affects individual and collective health^(7)^.

It is necessary to advance the discussion and production of educational games that are based on the *quilombola* teenager’s real needs and that contain scientific content to encourage the reader to criticize and reflect, as, in Brazil, 55.8% of the population declares itself to be black or brown^(8)^.

However, educational technologies for scientific dissemination in health commonly do not consider race/color components, making ethnic-racial inequalities invisible as a social determinant of adolescents’ experimentation in their rites of passage to the adult world. There are few publications on the use or consumption of alcohol in adolescence considering ethnicracial specificities^(9,10)^. Those available are developed in large urban centers, disregarding ethnic-racial diversity as part of the target audience, particularly those from rural *Quilombola* communities^(11)^.

In large part of innovation and game development, technical dimensions seem to be more emphasized than the participation of representations of the target audience in the production process. In this process, little space is given to debate the meanings, knowledge, and appreciation of human experiences between educators and students^(12,13)^, such as that between nurses and adolescents in the production of games related to topics of interest to health promotion. Therefore, recognizing the voice of adolescents in a *quilombola* community, building a dialogic and interactive educational material with Them and for Them can be a strategy for promoting life-protecting care.

The comic “Alcohol and adolescent rites in a *quilombola* community” (*Álcool e ritos de adolescentes em uma comunidade quilombola*)^(14)^ was elaborated with the participation of adolescents living in a *quilombola* community, in the north of the state of Espírito Santo, based on the daily experiences of alcohol experimentation as part of the rites of passage, common in the cultural festivals of the community, and in the intergenerational cohabitation. Girls and boys have lived with alcoholic beverages at home, in bars, and at parties at the community school, in the neighborhoods, at the football game, since a very early age in the socialization process.

Therefore, the study’s objective was to describe and discuss adolescents’ participation from a *quilombola* community in transforming the comic *“Possible Story” (Uma História Possível*), from the Comic *Álcool e ritos de adolescentes em uma comunidade quilombola,* into an educational game.

## METHOD

### Study Design

The participatory research was mediated by the art of the sensitive creative method (CSM) and developed in the community, having as structuring principles the collective decisionmaking, the approximation and distancing from the research field, and the negotiation of knowledge and cultural and community practices.

Artistic expression was the gateway to the human experience^(15,16)^, related to the way of life in the community, and allowed the production of knowledge focused on the participants’ social reality. The contribution of Freire’s philosophy in CSM lies in the fact that human beings, when reflecting on the world they live in, criticize it and act on it, transform their reality, create history, and produce culture^(13)^. In this regard, the creativity and sensitivity dynamics (CSD) are the guiding principle of CSM, since the artistic productions lead to critical and reflective group discussion anchored in the ideas that are the strengths of Freire’s philosophy^(16)^.

### Local

The field research was implemented from December 2018 to May 2019 in the rural area of the municipality of São Mateus, north of Espírito Santo, Brazil. In this area, surrounded by sugarcane and eucalyptus plantations, is located the *Quilombola* community, where a population that remains relatively semiisolated and distant from urban centers resides. The Basic Health Unit is 12 km from the community, and education is provided through a multi-teacher rural school, which covers primary education up to the 5th year of elementary school.

### Population

Adolescents who met the criteria of residing in the community, being enrolled in elementary school at the public school in the community, having preserved cognitive and motor skills and age between 10 and 19 years old, as defined by the World Health Organization^(17)^, were included. Adolescents with acute and chronic diseases were excluded.

For recruitment, the first meeting with parents or guardians of adolescents was scheduled for the second Sunday of December 2018, after the celebration of mass. Local leaders, parents, and adolescents attended the meeting, and the field researchers presented the research proposal and identified adolescents with the potential to participate as volunteers. The second meeting took place on the same day, at the community school, only with adolescents whose parents agreed to their participation, manifested by signing the Free and Informed Consent Form. Nine adolescents attended the meeting, six girls and three boys.

### Data Collection

It was developed in three group meetings when an awareness dynamic and two CSDs were implemented, as well as a meeting of field researchers to analyze and develop the storyboard and the material elaboration (two versions) by the designer and illustrator. The meetings were recorded with voice in MP3 and image using a portable digital camera.

At the first CSM meeting (first Sunday of December 2018), the awareness dynamics called “Shortening distances” was developed, with a duration of 30 minutes, aiming at an approximation between the research participants and the handling of materials (radio, camcorder, MP3 recorder, stationery, etc.) to be used in subsequent meetings. A bag of colored candies circulated within the group, and each person took one, while a music background harmonized the atmosphere of the environment. Then, each one looked for a pair with the same color of candy, sitting in a circle next to his/her pair, introducing him/herself to his/her pair with the generative question (GQ): “I’m (so-and-so) ... I’m (happy, willing...) I want to (do, create, produce)...” The individual presentations were relaxed and revealed that all the participants had blood ties, more specifically siblings and cousins. At the end of the activity, everyone agreed with the next day and time to meet again.

In the second meeting, which took place on the third Sunday of December 2018, the first CSD “Playing... transforming...”, was applied. A group of six adolescents participated, who were present at the sensitization meeting, four girls and two boys, and the meeting lasted approximately 50 minutes. In the first moment of the dynamics, during the participants’ welcoming, the materials (image projector, A4 paper, pens, pencils and eraser, dominoes, bingo, and board games) to be used in the dynamics were presented. In the second moment, the objective of the CSD was explained, and the GQ – ‘How Possible Story (*Uma história possível)*, from the Comic on Alcohol^(12)^, can be turned into an educational game?’ In the third moment, the image of *Possible Story* (*Uma História Possível)* was projected to allow following the story of a typical community character who consumed alcohol after a day of work on the field. Then, the investigators distributed three types of games (dominoes, bingo, and board) containing structured rules for the teenagers to try their application. The participants decided to form three pairs to engage in the games, discuss, and choose the one that would best fit the creation of a new game. The three pairs designed three artistic productions at the end of the moves. In the fourth and fifth moments, the pairs presented their (three) productions based on applying the rules of the games and the potential of each one so that the comic book *Uma história possível* ^(12)^ was adapted to the game language. The limits and possibilities rules, the purpose of each type of game, the specifics, and the spirit of the game within a particular time and space were questioned.

After this meeting, the field researchers kept out from the community for five months and met to analyze the adolescents’ images and narratives, which were recorded and transcribed, to prepare the first version of the design of the board game storyboard set by the adolescents.

In the second CSD (May 2019), which lasted two hours, “Shortening distances between what was produced and the educational game”, the group of five girls evaluated the preliminary storyboard. The first moment corresponded to the explanation about the CSD, its objective, the game (preliminary version), and the enunciation of the following GQ – “The information is... the rules are... the game is...” In the second moment, four adolescents participated in the board game and were observed by a fifth adolescent. The researchers just observed and recorded the participants’ comments, moves, and reactions. In the third moment, the group problematized what needed to be adjusted. At the last moment, the group synthesized its educational character in the game evaluation process, suggesting the points needing changes. Then, the materials produced at CSD were sent to a (hired) designer and illustrator for the graphic production and layout of the game’s final version.

### Data Analysis

Thematic analysis was adopted to classify, order, and systematize the empirical material^(18)^. Successive readings favored the immersion in the material to codify words and expressions in themes that gave rise to the content of the game cards and rules. The characteristics portrayed in the rural environment, people, and community composition were analyzed as fundamental to composing the game. The scientific and legal basis of the sections “did you know?” and “Trivia” was taken from the Comic^(14)^, being adapted to the board game cards.

### Ethical Aspects

The Research Ethics Committee approved this study of the Universidade Federal do Espírito Santo, Centro Universitário Norte do Espírito Santo, opinion No. 2.451.977, of 2017, following the provisions of Resolution No. 466, of December 12, 2012, of the National Health Council. The parents or guardians signed the Free and Informed Consent Form. At the end of the recruitment meeting, the Term of Assent was applied among the adolescents who remained in the room. Anonymization was adopted with the identification system of the acronym AD for adolescents; M or F, for male or female; and 1, 2, 3... for the sequence of narrative intervention in the dynamics (ADM1, ADF2...).

## RESULTS

The number of adolescents varied in the meetings (first meeting, nine – three boys and six girls; in the second, six – two boys and four girls; and in the third, five girls). They were aged between 11 and 15 years (one boy aged 11 and two aged 13; one girl aged 12, two girls aged 13, one aged 14, and two aged 15), eight were Catholic, and one was Evangelical. All of them attended elementary school, three in the multi-teacher school in the community (from the third to the fifth year) and six in the *Escola Família Agrícola* (from the sixth to the ninth year) located outside the community. They all lived in a family with a monthly family income below the minimum wage.

### Participation in the Creation of the Board Game

**Figure 1:**
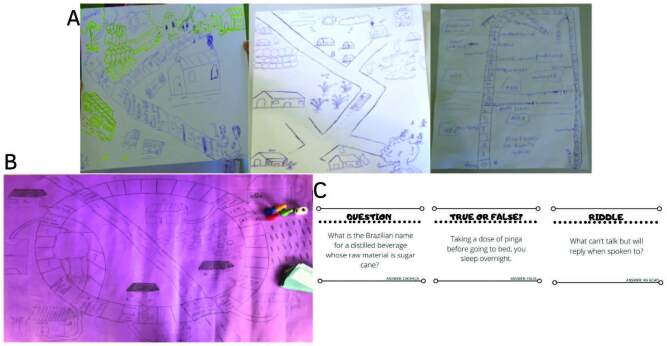
Mosaic of group artistic productions by girls and boys (A). CSD “Playing... transforming...” in the composition of the board game (B)preliminary storyboard and cards (C) elaborated by the researchers, based on images and narratives of the adolescents who participated in the CSD. São Mateus, Espírito Santo, Brazil, 2019.

After the group’s internal debate and negotiation, the adolescents decided on the board game as the most appropriate to represent the comics. The mosaic of artistic productions (Figure 1A) represents the need for the game to portray the community where they live (their houses, church, bars, plantations, *gameleira* tree, river), creating an info geographic and cultural identity. In the figure, the board game background is visualized with the squares along the paths to demarcate “START” and “END”. There are obstacles to challenge the player when rolling the die, indicating the move forward or backward on the squares.

The narratives of adolescents in the presentation of artistic productions.

*At the beginning of the Board Game, the image of gameleira tree, a path that goes to the bar, and another going back to the family’s house. Here,* [pointing to the image], *is the start, with the plantations and the river*. *On the way to the family home, he passes by the school. At the house door, the mother and children are waiting for him. At the house entrance, there will be challenges Up there, there are two paths, you roll the die, and the die will decide. If number two falls, you move forward. In the challenges, there is an option to go forward or backward.* (ADF1, ADM2, ADF5)

The group decided that the board game would have the same name as the comic *Uma história Possível* ^(12)^, aimed at the target audience aged between 10 and 19 years, with up to four players being able to engage. The game pieces included a die numbered from 1 to 6, four differently colored pawns, rules, and cards with true or false statements, questions, and riddles with the contents.

The rules established were based on the narratives of the adolescents who participated in the CSD “Playing... transforming...” to adjust the behavior and organize the actions of each participant in the game. The first version of the board storyboard with the cards is shown in Figure 1 (B and C), with the following structure of rectangles, called “square”. A “START” square – seven squares directing the “CARDS” – True or False, Questions and Riddles – about the alcoholic beverage to be problematized; eight “TRAP” squares that indicate “DON’T PLAY FOR A TURN”; three bars selling drinks to indicate the “BACK TO START,” one “SHORTCUT” and another for the “END” of the game. The board game cards included information extracted from the comic book script, the scientific content of the “True or False” and “Did you know” sections of the Comic *Álcool e ritos de adolescentes em uma comunidade quilombola* ^(14)^.

**Figure 2:**
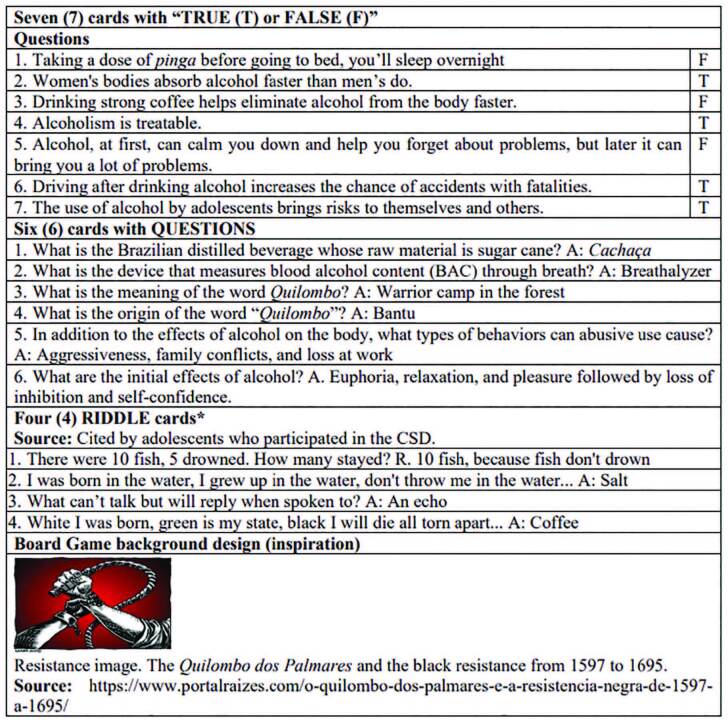
The cards content of the Board Game “Possible Story”(Uma História Possível).

Of the 17 cards, seven corresponded to myths, treatment, consequences, and vulnerabilities for players to assign “TRUE or FALSE” to the statements. Six “QUESTIONS” included substance types, the measurement of blood alcohol content (BAC), consequences, and effects of drinking alcohol in the community. Four “RIDDLES” did not contain information about alcohol, but the intention when keeping them was to create an identity of the educational material with the target audience for which it was intended (Figure 1C and Figure 2).

Adolescents had produced the image of the game path with a straight line (Figure 1A); the team of investigators proposed a circular path in the first version of the storyboard (Figure 1B); but, after consulting the *Portal Raízes*, the game’s path was changed to bring it closer to the history of Afro-descendant ancestors and their culture. Thus, an inserted image (Figure 1) depicting the black resistance at the time of slavery serves as an inspiration for redesigning the path of the Board Game. The arms’ symbol was transformed into paths and the whip into a river.

### Board Game Evaluation by the Target Audience

As for the board design presentation, the adolescents evaluated that its layout expressed the *quilombola* community where they lived. However, they suggested changes in the game’s operating rules.

*The board image shows what is in our community. There shall have more questions on the board. In the shortcut and the end, there has to be a question.../Whoever answers a question wins. Always go two rounds without playing* //*When it lands on T (true) or F (false), there must be an explanation to continue the game* (ADM2).

*It is better to decrease the number of rounds without playing* (ADM1 and ADM3).

*Go a square backward if you don’t get it right* (ADF4).

*To win, you have to get the number on the die and still answer the question. If you make a mistake, go back one square only, keep trying until you win* (ADM3).

Regarding the general aspect of the board game, the group of adolescents considered it:

(...) *funny, awesome!... cool; it makes me want to keep playing and take it home* (ADM1 and ADM2).

**Figure 3:**
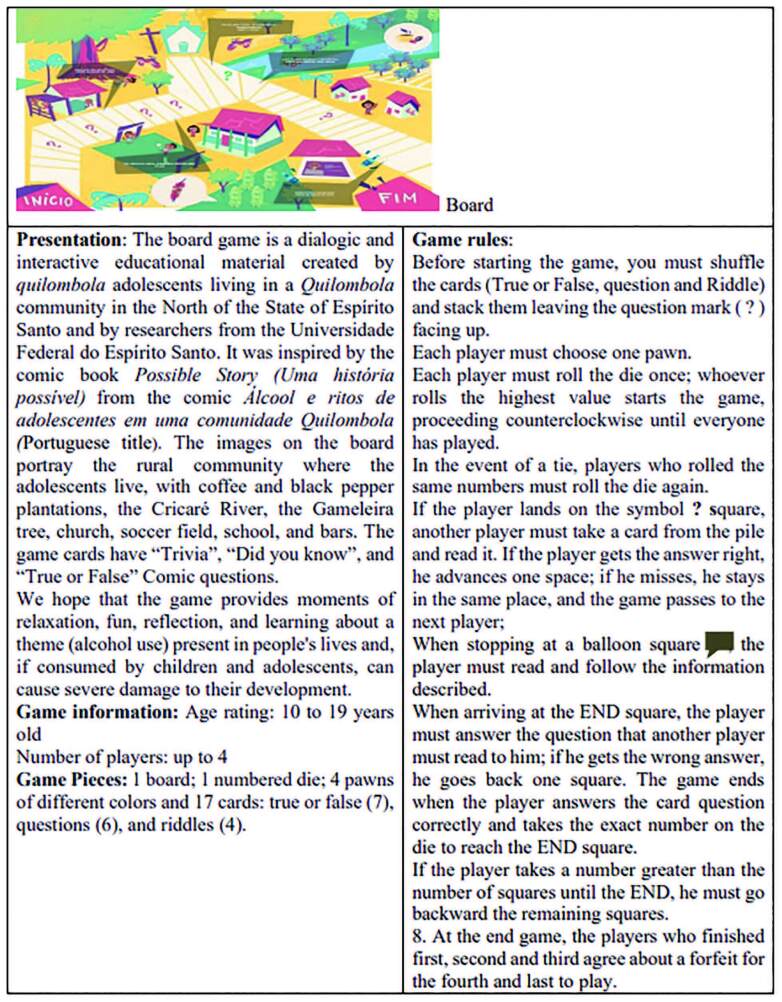
Board Game Possible Story (Uma história possível). São Mateus, Espírito Santo, Brazil, 2020.

After evaluation by the adolescents, the rules were adapted to this new path, with the addition of topics: presentation, information, material that makes up the game, and game rules (Figure 3). The illustrator designed the new version to compose the educational game with 17 cards.

## DISCUSSION

The participation of adolescents from the *Quilombola* community in the development of a board game about alcohol consumption recognizes this group’s voice as the educational game’s target audience. It was a participatory process involving engagement, democracy, and interactivity for transforming a comic into a game whose central theme is alcohol consumption, a sensitive theme to be addressed in promoting health in adolescence.

Educational materials for adolescents from the *Quilombola* community, as the representatives of a group with ancestors from the African continent, are scarce^(19)^. Regarding the various topics of interest for promoting adolescent health, there is a lack of information considering the ethnic-geographic components that allow them to identify readers with characters, scenarios, and social context. This group is on the sidelines of access to health information, as educational materials have reflected more frequently the ontological view of the educator and the information producer; instead of producing knowledge, it constitutes a cultural invasion^(20)^. The adolescents were able to share their experiences in constructing and evaluating this material.

The participatory nature, based on the community and art, favored the production of technology, uniting health professionals, researchers, and specialists with users in the process of co-creation. In the group discussions, the adolescents exposed their opinions, beliefs, and values and actively listened to the different perceptions that stimulated new ideas. When invited to participate in the co-creation process, young people focus on developing virtual reality, feel included, empowered, and able to generate new ideas^(21)^. Educational materials are of great importance in the teaching-learning and health promotion process, as they are qualified as facilitators of learning and not just as an informational resource^(22,23)^. Board games are an alternative to traditional teaching-learning educational methods in acquiring knowledge and making decisions^(24–26)^.

Scientific evidence indicates that most educational games produced on drugs (including alcohol) have not yet managed to overcome the biomedical vision, encouraging the person to memorize and condition^(1,4–6,19,21,23,25)^. Scientific updating and the incorporation of creative and critical educational content in educational games, and the incorporation of the ethnicracial character^(11)^ are required. The board game produced in this study is an exciting example of the union of cultural and ethnic/racial aspects with thoughtful criticism so that health education is emancipatory.

Alcohol consumption is one of the most complex public health problems in adolescence due to the greater vulnerability of this age group to the influences of socio-environmental stimuli. Habits and behaviors are constantly changing, increasing adolescents’ exposure to vulnerabilities^(27)^. Especially for adolescents living in rural areas, there is more incredible difficulty accessing services and information. Moreover, fewer studies are available pointing out the real problems affecting coping strategies that rely on intersectoral articulation (health and education) and partnerships with civil society.

The group of adolescents who live in *quilombola* communities is culturally and geographically diverse in Brazil, with social and economic inequalities based on a subsistence economy of land exploration. This reference was recognized and is printed on the board game for its graphic representation. Adolescents who live in the context of a *quilombola* community are distinguished from those belonging to other races and ethnicities of Caucasian origin and other social classes. Getting adolescent among individuals living in *quilombos* is based on traditional principles with conservative habits and customs of African ancestry. In this sense, values and behaviors have a family matrix and are reproduced intergenerationally with the reinforcement of the entire community. In addition to the aspects mentioned above, a review study, which does not consider ethnic-racial aspects, suggests that the media positively influences the increase in alcohol consumption, the increase in excessive alcohol consumption, and dangerous drinking behavior^(28)^; however, these aspects were not present in the comic, and therefore were not considered by the teenagers in the present game.

As a limitation of the study, there is the high cost of printed material to make it available to the target population, as internet access in the rural community is restricted. In addition, the study was developed in a *Quilombola* community, hindering the generalization of data to contexts that do not present similar sociocultural conditions.

The implications were that there was a creative process of a board game, a technological resource of health care that facilitates and guides the discussion of relevant topics, such as alcohol abuse. In health care, the game can be applied, as far as the specificities of the demands and needs of these adolescents are respected, focusing on inclusion and equity to overcome social inequalities, which remain in the field of health. One way to counteract the cultural invasion of colonizing Caucasian ethnicities to Afro-descendants is to promote the union of local cultural knowledge with scientific knowledge, particularly concerning alcohol consumption, as it is a topic in an educational material that favors scientific dissemination, allowing adolescents to reflect on the world in which they live.

## CONCLUSION

The engagement of adolescents, who live in a *Quilombo*, took place from the conception to the evaluation of the board game, with playful potential for developing health promotion and preventing diseases. Furthermore, they were encouraged to reflect on a community context of cultural permissiveness of alcohol use, mainly due to the ethnic-racial characteristics of Afro-descendant residents in *quilombos*, a social group little remembered in the production of games.

The sensitive-creative method was strategic for the adolescents from a *Quilombola* community to express their reflexive criticism anchored in the design of their community as a form of artistic expression in the creation of the game. The methodology of transforming a comic book into an educational game about alcohol consumption was a core issue and also recognized adolescents’ voice and active participation from a *Quilombola* community.

## ASSOCIATE EDITOR

Cássia Baldini Soares
